# Preface to the theme issue ‘Durability and ageing of composite materials'

**DOI:** 10.1098/rsta.2021.0224

**Published:** 2023-01-09

**Authors:** Marco Gigliotti, Jean-Claude Grandidier

**Affiliations:** ^1^ Department of Physics and Mechanics of Materials, Institut Pprime CNRS, ISAE-ENSMA, University of Poitiers, France; ^2^ ISAE-ENSMA Téléport 2–1 avenue Clément Ader BP 40109 86961 Futuroscope Chasseneuil, France

**Keywords:** composite materials, durability and ageing, experimental and numerical methods, fatigue, multiphysical couplings, multi-field analysis

## Abstract

The issue focuses on ageing and durability of composite materials for industrial applications. Ageing and durability are two fundamental topics within the context of design and optimization of materials and structures: ageing may induce degradation and leads to premature failure. Therefore, understanding the main ageing mechanisms is of paramount importance for predicting material durability. These topics are still barely studied systematically and the main related issues are debated within mechanical, chemical, and solid-state physics research communities. This issue aims to update the current research state of the art, clarifying the interplay between chemical and physical mechanisms involved in degradation processes, and providing test protocols and design rules that account for ageing and durability.

This article is part of the theme issue 'Ageing and durability of composite materials'.

The optimal design of transport and energy structures requires the best choice of materials and processes according to the specifications of each application. Owing to their high specific properties (stiffness-density ratio, strength-density ratio) and good fatigue behaviour, polymer matrix composites are widely used and their field of application has been growing over the last 50 years. In the context of this synergy, engineers are faced with the need to increase their knowledge and thus control the ageing phenomena of composites and more particularly of the polymer matrix, especially at high temperature and in humid environments [1–5].

The establishment of design protocols integrating all these phenomena is of paramount importance in the coming decades, and research work must lead to specific characterization tools and theoretical models capable of understanding and describing the multiphysical and multiphase couplings. The development of efficient, robust and faithful numerical tools is also part of the challenge, as virtual design is now part of industrial uses.

As the intrinsic complexity of this subject suggests many challenges of a different nature, both scientific and technical, the community has therefore largely focused on the multi-physics and multi-scale aspects of ageing in order to accurately predict the durability of materials and structures. In the literature, research work deals with the different aspects of this problem, and taking advantage of the latest advances is essential to bring out new questions, future themes to be developed and figure out the experimental and numerical tools to be built.

This aim of the present theme issue is to bring together several fields of study that are all of paramount importance when assessing the issues of durability and ageing of complex materials: chemistry, physics, materials science, mechanics, computational methods, numerical simulations, identification and optimization. Chemistry and physics are at the basis of knowledge by characterizing the primary mechanisms of degradation related to the ageing of materials: the diffusion of species, their mutual reactions and the chemical effects induced on the kinetics of non-equilibrium processes and on thermomechanical properties. As such, materials science is at the forefront of evaluating the effect of chemical species on these properties at different scales.

Mechanics of materials is necessary to take into account the effect of degradation on the main mechanical fields (deformation, stress). Faced with a non-equilibrium situation and in the presence of multiple gradients, computational mechanics is necessary or even mandatory to simulate the complexity of the scenario described and to understand and identify it. Experimental and numerical methods must often be carried out in synergy and addressing these two items is one of the keys to increasing knowledge. In the context of composite structures, the multi-scale and multi-physics character of material degradation is to be understood to predict the durability of complex structures.

In particular, this theme issue first explores some topics concerning the characterization and modelling of the effects of degradation and ageing on the physico-chemical properties of polymeric materials, such as changes in the glass transition temperature of polymeric matrices exposed to low molecular weight penetrants, as well as the hygro-viscoelastic behaviour of polymer-based composites used in marine environments. In order to account for these effects in composites, the multi-scale and multi-field character of degradation modelling is illustrated through the presentation of global-local multi-field models for stress analysis of laminated structures exposed to environmental solicitations. The topics of material degradation and durability are explored through the characterization and prediction of the relationship between translaminar fracture toughness and pull-out length distributions in composite materials exposed to high temperatures, the modelling and simulation of fatigue crack propagation by a peridynamic approach for composite materials, a detailed account of fatigue phenomena in fibre reinforced hybrid plastics.

This issue should have a significant impact both within the academy (at least in the groups working in this field) and in the mechanical industry.

## Data Availability

This article has no additional data.
